# Deletion of RBP-Jkappa gene in mesenchymal cells causes rickets like symptoms in the mouse

**DOI:** 10.1007/s44194-022-00007-w

**Published:** 2022-05-26

**Authors:** Yan Gao, Jemma Victoria Walker, Christopher Tredwin, Bing Hu

**Affiliations:** Stem Cells & Regenerative Medicine Laboratory, Peninsula Dental School, Faculty of Health, University of Plymouth, 16 Research Way, Plymouth, PL6 8BU UK

**Keywords:** Notch, Bone, Cartilage, Wnt5a, Rickets

## Abstract

Crosstalk between different signalling pathways provide deep insights for how molecules play synergistic roles in developmental and pathological conditions. RBP-Jkappa is the key effector of the canonical Notch pathway. Previously we have identified that Wnt5a, a conventional non-canonical Wnt pathway member, was under the direct transcriptional control of RBP-Jkappa in dermal papilla cells. In this study we further extended this regulation axis to the other two kind of skeletal cells: chondrocytes and osteoblasts. Mice with conditional mesenchymal deletion of RBP-Jkappa developed Rickets like symptoms. Molecular analysis suggested local defects of Wnt5a expression in chondrocytes and osteoblasts at both mRNA and protein levels, which impeded chondrocyte and osteoblast differentiation. The defects existing in the RBP-Jkappa deficient mutants could be rescued by recombinant Wnt5a treatment at both cellular level and tissue/organ level. Our results therefore provide a model of studying the connection of Notch and Wnt5a pathways with Rickets.

**Supplementary Information:**

The online version contains supplementary material available at 10.1007/s44194-022-00007-w.

## Introduction

Notch pathway is a highly conserved signalling pathway for cell fate control. Mouse models with defects or overexpressing Notch signalling have been connected with different skeletal pathogenesis (Regan and Long 2013; Zieba et al. 2020). During the past two decades, our knowledge for understanding of Notch’s functions in osteoblasts and chondrocytes have been ever enriched (Canalis 2008; Fujimaki et al. 2006; Mead and Yutzey 2009; Nakanishi et al. 2007; Sassi et al. 2009; Schnabel et al. 2002; Sciaudone et al. 2003; Shimizu et al. 2007). The Wnt pathway is another well-established pathway that can regulate chondrocyte and osteoblast fates as well as craniofacial development (Church et al. 2002; Geetha-Loganathan et al. 2009). Crosstalk between Notch and Wnt pathways have been reported during development (Clevers 2013; Doupe et al. 2010; Hu et al. 2010; Lim et al. 2013; Lowell et al. 2000; van Es et al. 2005) and cancers (Bolos et al. 2007; Clevers 2006). In the skeletal system, the connection of Notch and Wnt are still not clear although a recent report has suggested encouraging connection of the two pathways in bone wound healing (Lee et al. 2021).

Among all the Wnt family numbers, Wnt5a, a typical “non-canonical” Wnt family member is one of the mostly well-studied molecules that have been shown to be able to regulate chondrocyte and osteoblast development (Yamaguchi et al. 1999; Yang et al. 2003). Mice with complete deficiency of Wnt5a developed significant skeletal development defects. e.g. mice with homozygous deletion of Wnt5a die during embryonic stage with significant delay of bone development and enlarged hypertrophic zone in the long bones, while in the heterozygote, a decrease of bone density in the adults have been reported (Kawakami et al. 1999; Liu et al. 2008). Previously we have identified direct transcriptional regulation of Wnt5a by RBP-Jkappa, the key Notch pathway transcriptional factor, in one fraction of functional dermal cells: dermal papilla cells (Hu et al. 2010). In this study, we further illustrated this regulation axis connection could be extended to skeletal system where deletion of RBP-Jkappa caused Rickets like symptoms that could be partially rescued by Wnt5a treatment.

## Results

### Mesenchymal RBP-Jkappa deletion caused rickets like symptoms

**Fig. 1:**
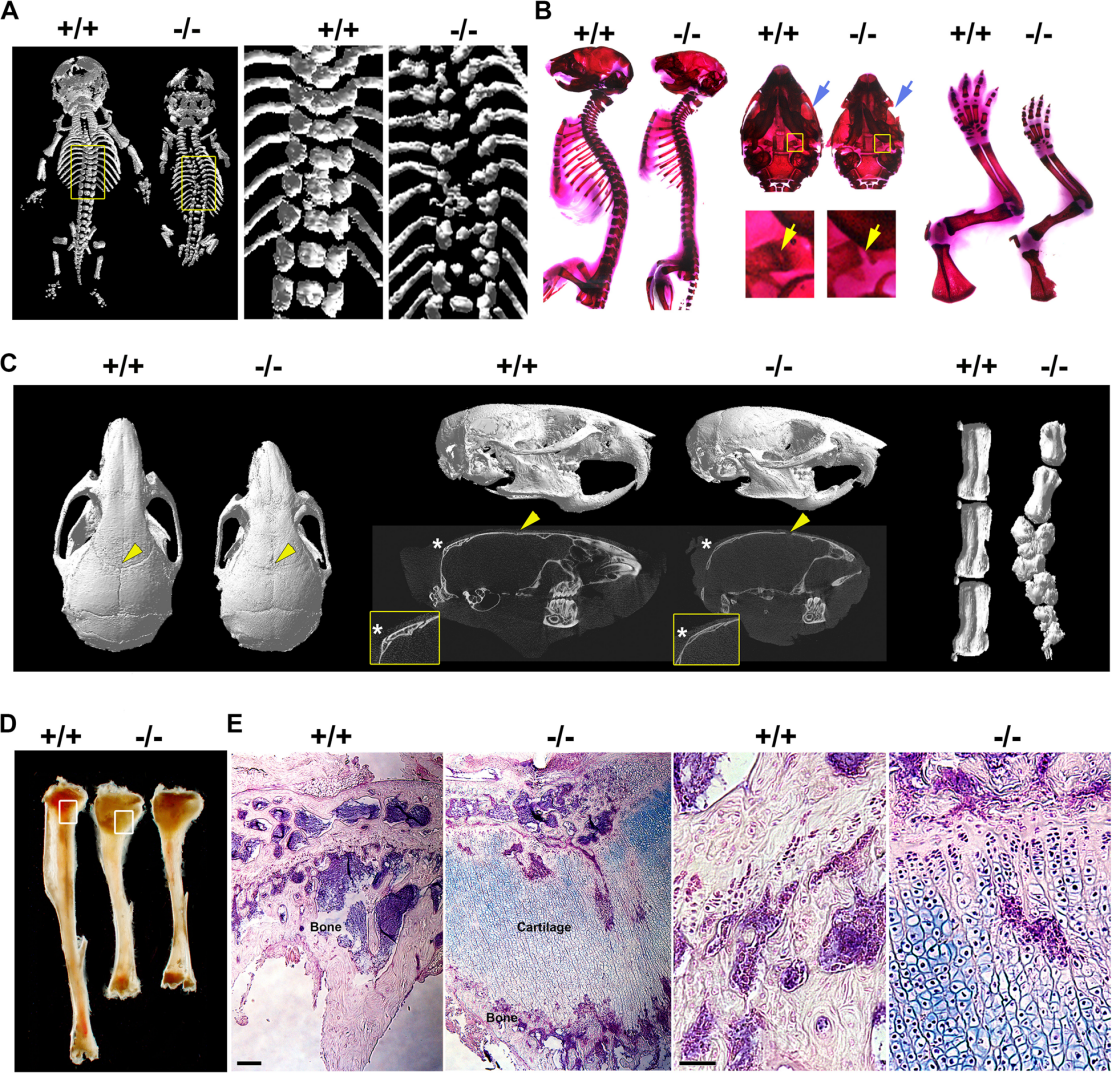
Losing RBP-Jkappa in skeletal system in the mice induced Rickets like symptoms. **A** Represented Micro CT analysis of postnatal day 0 (P0) litter mate male control (RBP-Jkappa loxp/loxp, referred as +/+ thereafter for all the legends) and male Collagen 1 a2 Cre x RBP-Jkappa loxp/loxp, referred as −/− thereafter for all the legends). Right two panels represent the squared regions in the left panel. **B** Represented Alizarin Red staining of the P6 mice. Left panel: fore limbs were removed to better view spinal curvature. Middle two panel: arrows marked the compared region of ossification in zygomatic arches and skull bases. Right two panel: comparison of the fore limbs. **C** Micro CT analysis of the 4 weeks old mouse skulls. Right panel: top views. Middle panel: right lateral views. Stars marks parietal bones. Arrows indicate the sutures at the junctions of nasal and frontal bones. **D** Stereo view of tibias from 4 months old mice. **E** Alcian Blue and eosin staining of the tibias showed in the regions marked by white rectangles in **D**. Bars: E and F: 100 μm

We utilized Collagen 1 a2 Cre mice to target mesenchymal cells including osteoblasts and chondrocytes (Florin et al. 2004). After we crossed this Cre line with RBP-Jkappa loxp/loxp mice (Hu et al. 2012; Hu et al. 2010), we observed a significant fraction of these mice (> 20%, 20 out of 79 KO pups) died immediately after birth. Analysis of these mice by computerised Tomography (micro CT), revealed profound delays in skull ossification, defective vertebrae fusion, winged scapulae, and short limb formation (Fig. 1A). Whole mount analysis of the skeletal system of other mice that survived until 6 days after birth showed a lack of spinal curvature, defective skull and zygomatic arch ossification and shorter limbs (Fig. 1B). Even less affected mice at 4 weeks of age exhibited incomplete closure of the cranial suture, reduced bone thickness and density, and abnormal tail vertebrae (Fig. 1C). The phenotype became more severe at 4 months, when long bones stop growing. The shorter radius and tibia of mice with the RBP-Jkappa −/− deletion had an expanded region of chondrocyte proliferation and hypertrophy that had not been replaced by bone structures (Fig. 1D and E). The phenotypes developed in the (ColIa2-Cre x RBP-Jkappa loxp/loxp) mice therefore simulate Rickets symptoms that have similar skeletal deformities (Narchi et al. 2001).

### Skeletal defects in the ColIa2-Cre x RBP-Jk loxp/loxp mice are due to the defects of chondrocytes hypertrophy and apoptosis, and defects of osteoblasts proliferation and differentiation

**Fig. 2:**
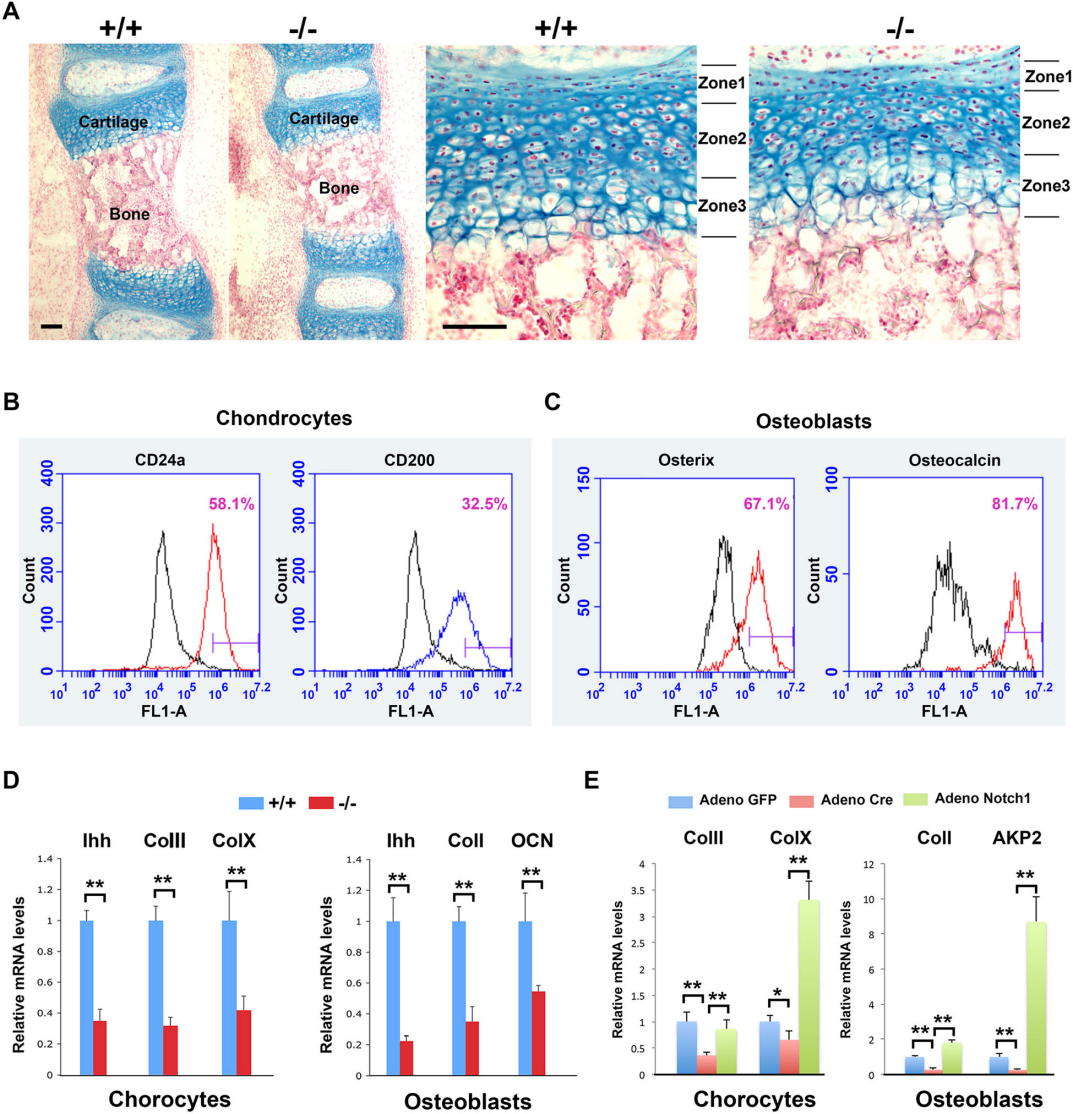
Key chondrocyte and osteoblast differentiation markers are under the control of Notch and RBP-Jkappa. **A** Representative Alcian Blue and nuclear fast red staining of the vertebra at postnatal day 0. Note the significant reduce cartilage layers: Zone 1: Superficial zone, Zone 2: Transitional zone, and Zone 3: Radial zone. **B** and **C** Flow cytometry analysis of chondrocyte markers (CD24a and CD200) and osteoblast markers (Osterix and Osteocalcin) on +/+ cells used in the study. **D** Real time RT-PCR analysis of the indicated genes expressed in the freshly isolated chondrocytes and osteoblasts using specific primers from P10 mice. **E** Gene expression analysis using Adeno virus meditated Cre and Notch expression on the +/+ chondrocytes and osteoblasts. ** *P* < 0.01. Abbreviations: Ihh: Indian Hedgehog; Col1: Collagen I; ColII: Collagen 2; ColX: Collagen X; AKP2: Alkaline Phosphatase. Bars: A and B: 40 μm

We next performed examination of the vertebrae of the mutants at postnatal day 0. The RBP-Jkappa deficient mice already have reduced ossified bone length, as well as the reduction of cartilage stratification zones (superficial, transitional and radial zones) (Fig. 2A). These suggesting the defects on skeletal development in the RBP-Jkappa knockouts already happened before birth.

To confirm the skeletal changes were due to endogenous changes, we isolated chondrocytes from tibia cartilage postnatal day 10 control mice and 2 week old mouse parietal bones. Flow cytometry analysis using chondrocyte markers: CD24a and CD200 (Fig. 2B) (Belluoccio et al. 2010), and osteoblast markers: Osteorix and Osteocalcin (Fig. 2C) (Eghbali-Fatourechi et al. 2007) suggested successful isolation and enrichment of the two cell populations. Real time RT-PCR analysis suggested a significant down-regulation of chondrocyte differentiation and hypertrophy markers: type II and type X collagens, already exist at this stage, as well as a key molecule in chondrocyte development: Indian Hedgehog (Ihh) (Fig. 2D). In parallel, in the osteoblasts, Collagen I and Osteocalcin expression as well as Ihh expression were highly down-regulated (Fig. 2E).

To understand if the down-regulation of these two markers were due to the intrinsic/local effects in the chondrocytes after RBP-Jkappa deletion rather than systematic defects, we cultured the chondrocytes and osteoblasts from RBP-Jkappa loxp/loxp mice and infected cultured cells using either Adeno Cre or Adeno-Notch1 viruses (Hu et al. 2010), the results showed in chondrocytes a significant up regulation of Collagen X, but not Collagen II, suggesting Collagen X is the direct target of Notch-RBP-Jkappa, while the rescue of Collagen II might be a secondary effect (Fig. 2E). Whilst in osteoblasts, Collagen I and AKP2 were efficiently down regulated by removing the RBP-Jkappa gene and up regulated upon Notch1 viral induction (Fig. 2E).

### Skeletal defects of ColIa2-Cre x RBP-Jkappa loxp/loxp mice are linked with Wnt5a deficiency

**Fig. 3:**
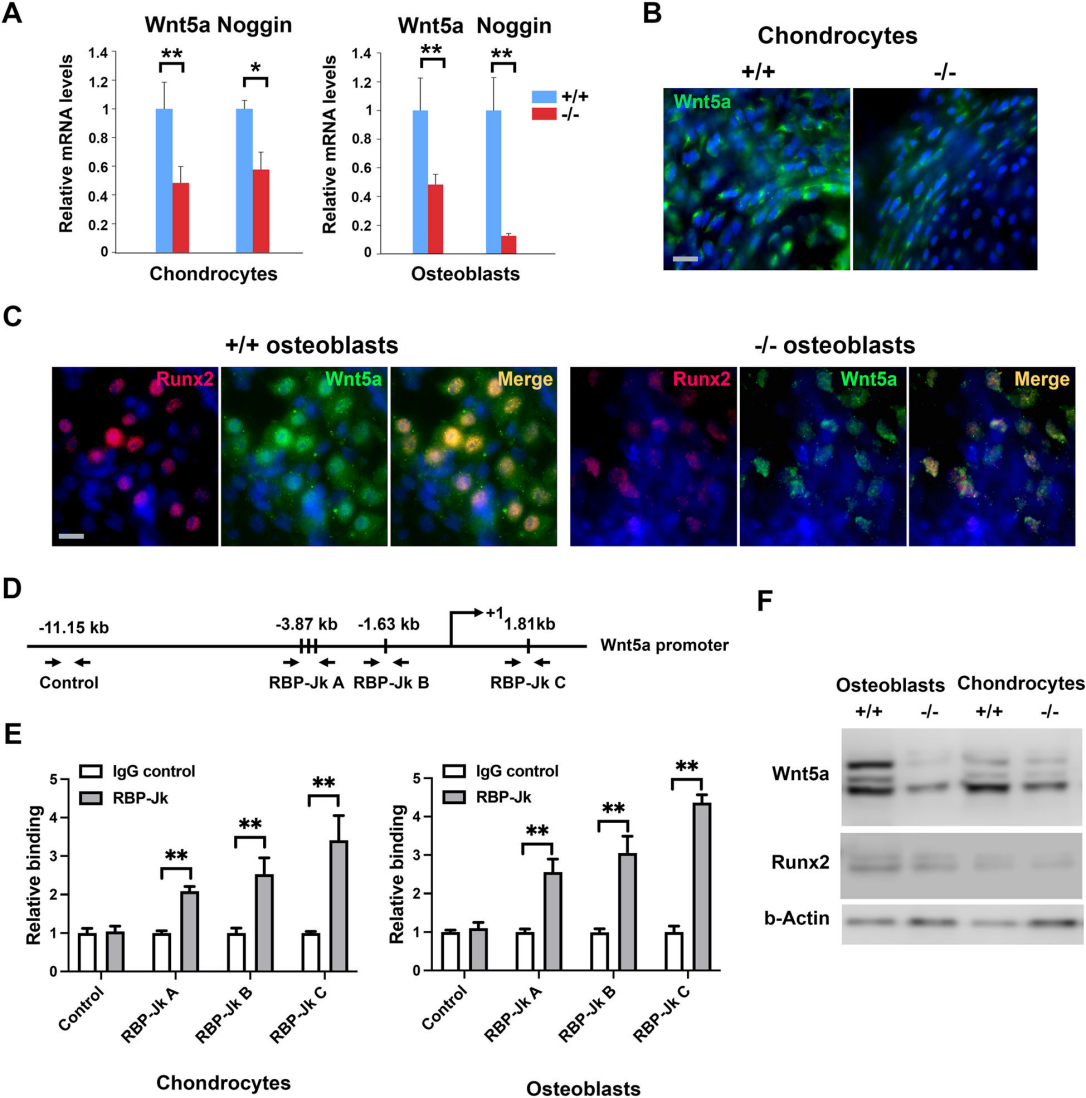
Wnt5a is down-regulated in the RBP-Jkappa deficient chondrocytes and osteoblasts. **A** Real time analysis of Wnt5a and Noggin in the in the freshly isolated chondrocytes and osteoblasts using specific primers. * *P* < 0.05, ** *P* < 0.01. **B** Immunofluorescence analysis of Wnt5a (green) in the mesial tibia remote growth plate of the P10 mice. **C** Runx2 (red) and Wnt5a (green) expression analysis in the tibia remote ossification regions close to the tibia growth plates showed in **B**. **D** Predicated RBP-Jkappa bind sites at the mouse Wnt5a promoter. Region A (RBP-Jk A) has 3 predicated binding sites, and regions B and C have two sites. Control: where the control sequence of no RBP-Jkappa binding site were selected for internal control. **E** Chromatin IP analysis of RBP-Jkappa to the indicated binding sites showed in **D**, in +/+ cells. **F** Western blotting analysis of Wnt5a and Runx2 expression in the indicated cells. The same membrane was plotted sequentially with the indicated antibodies. Bars: 10 μm

Similar skeletal alterations, such as defective vertebrae fusion and bone development have been described for mice with deletion of the Wnt5a gene (Yang et al. 2003). And recently we have found that in one fraction of mesenchymal cells, dermal papilla cells, Wnt5a is under direct control of Notch-RBP-Jkappa (Hu et al. 2010), we hypothesize that in the skeletal system, the same regulation axis might still exist. Indeed, real time RT-PCR analysis of chondroblasts and osteoblasts freshly isolated from P10 mice with the RBP-Jκ deletion confirmed the decreased Wnt5a expression, and also Noggin that we found were down stream target of Wnt5a in dermal papilla cells (Hu et al. 2010) (Fig. 3A). The expression of Wnt5a protein, as assessed by immunofluorescence analysis, was strongly down regulated in the tibia chondroblasts of mice with the RBP-Jκ deletion (Fig. 3B), as well as in osteoblasts of the bone growth region of those mice (Fig. 3C).

Chromatin IP analysis on primary control chondrocytes and osteoblasts suggested that as transcriptional factor, RBP-Jkappa indeed also bind to the predicted binding sites (Fig. 3E), similar to what we previously reported in dermal papilla cells (Hu et al. 2010). Western blot analysis also confirmed in the isolated primary cells, Wnt5a and Runx2 were both down-regulated in the osteoblast and chondrocytes (Fig. 3F).

### Notch-RBP-Jkappa regulates chondrocyte hypertrophy and apoptosis, osteoblasts proliferation and differentiation through Wnt5a

**Fig. 4:**
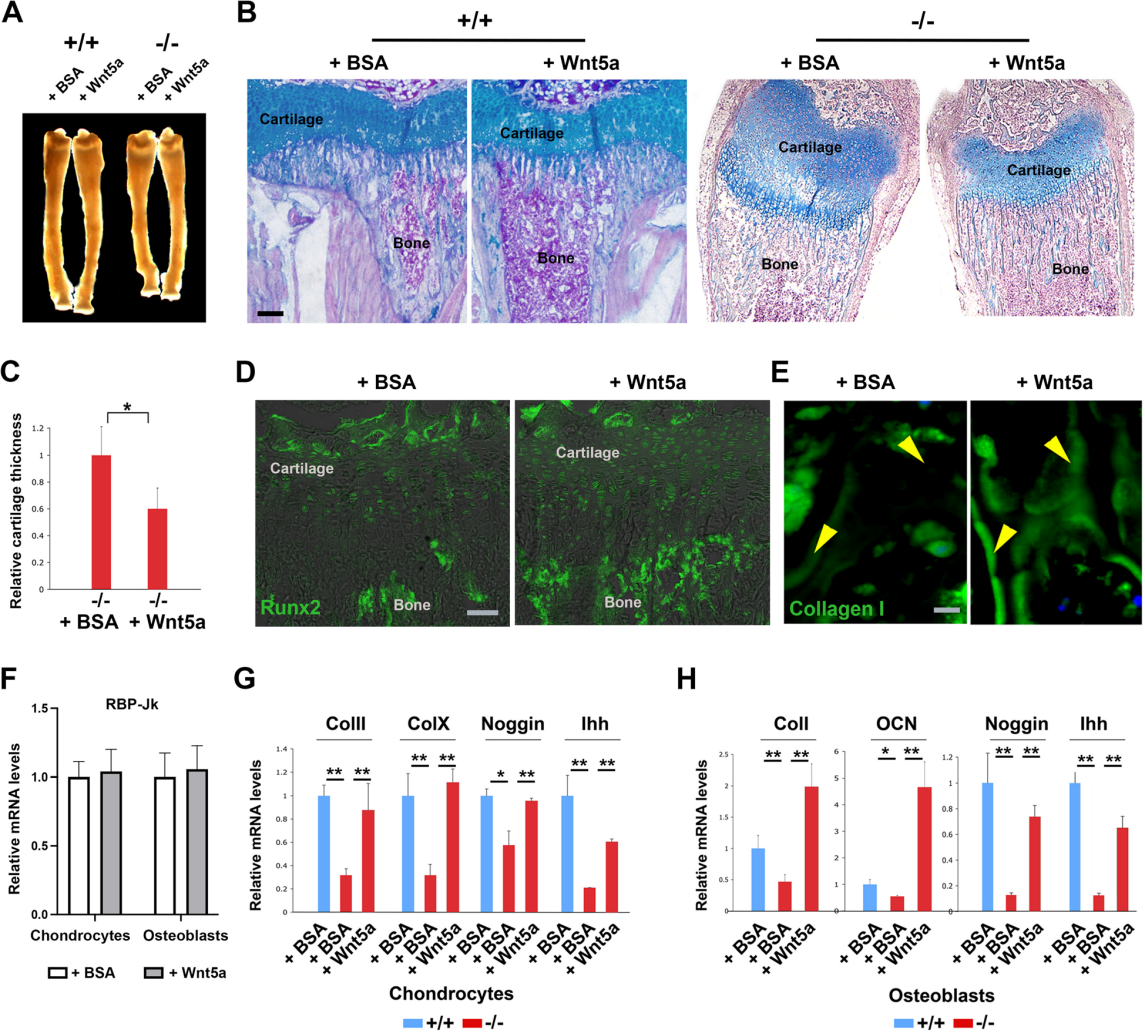
Wnt5a could rescue skeletal phenotype in the RBP-Jkappa deficient mice. **A** Representative stereo images of the tibia organ culture (*n* = 3 pairs for each condition, with left tibia used for BSA and right for Wnt5a treatment). **B** Represented images of Alcian Blue and nuclear fast read staining of paired P10 −/− tibiae organ culture either treated with BSA or Wnt5a for 7 days. The mesial ends of tibiae cultured in the indicated conditions as showed in **A** were used for analysis. **C** Cartilage thickness measurement in the −/− samples showed in **B**. **D** Immunofluorescence analysis of Runx2 (together with phase-contrast filter) expression in the samples showed in **A**. Note that Rux2 expression significantly increased in the Wnt5a treated samples. **E** Collagen I immunostaining on the distal ossification region near growth plate in the same samples showed in **B** and **D**. Arrows mark bone trabecular surface. **F** Real time RT-PCR analysis of RBP-Jkappa expression in the +/+ cells treated with or without Wnt5a. **G** and **H** Real time RT-PCR analysis of the indicated genes expressed in the freshly isolated chondrocytes and osteoblasts treated with indicated conditions, using specific primers. * *P* < 0.05, ** *P* < 0.01. Abbreviations: Ihh: Indian Hedgehog; Col1: Collagen I; ColII: Collagen 2; ColX: Collagen X; AKP2: Alkaline Phosphatase. Bars: B and D: 100 μm; E: 10 μm

To assess whether the defective ossification is linked to decreased Wnt5a expression, tibiae from mice with the RBP-Jkappa −/− deletion were placed in culture for several days with/without exogenous Wnt5a (Fig. 4A). We found that while Wnt5a had no obvious effects on control samples (Fig. 4B and data not shown), addition of Wnt5a could cause a significant reduction of the chondrocyte proliferative/hypertrophic zone together with an enhanced zone of ossification in the RBP-Jkappa deficient mice (Fig. 4B and C). That was accompanied by increased Runx2 expression in the cells within cartilage and bone (Fig. 4D), and as well as Collagen I deposition (Fig. 4E). While Wnt5a on chondrocytes and osteoblasts from control mice did not affect RBP-Jkappa transcription (Fig. 4F). Therefore, indeed Wnt5a is a downstream effector of RBP-Jkappa in controlling bone growth. Finally, when placed in culture, isolated RBP-Jkappa −/− chondrocytes and osteoblasts showed strongly decreased expression of differentiation markers (Collagen II, Collagen X, Noggin and Ihh for Chondrocytes, and Colllagen I, and Osteocalcin for Osteoblasts), which were efficiently rescued by Wnt5a treatment (Fig. 4G and H).

## Discussion

Significant information has been known on the role of the Notch pathway in cartilage and bone development. In particular, Notch1 mutant mice undergo disorganized somitogenesis (Swiatek et al. 1994); mutation of Notch2 can cause progressive bone loss (Simpson et al. 2011); and blockage of Notch signalling or knockdown of Notch cause a suppression of chondrocyte proliferation and associated differentiation (Karlsson et al. 2008; Karlsson et al. 2007). However, little is known of the mechanisms underlying Notch function in this context. RBP-Jkappa, the key transcription factor of the Notch pathway, sits on the promoter of many target genes. Although in general, RBP-Jkappa is considered as a down stream target suppressor in the absence of Notch receptor cleavage and nuclear translocation, the evidence was largely achieved in Drosophila (Kopan and Ilagan 2009). In the chondrocyte and osteoblasts, how RBP-Jkappa meditates their biological activities were not known before our study. We found that RBP-Jkappa is important both for chondrocyte differentiation and hypertrophy as well as osteoblasts differentiation not only in the cultured cells, but also in the ex vivo cultured explants.

Importantly, the deletion of RBP-Jkappa in the mice, has evoked Rickets like symptoms, which made the investigation far more exciting as so far not much has been known of which gene pathways are involved in this disease beside Vitamin D receptors (Pettifor 2004) and Fgf23 (Consortium, A 2000). Recently one report has shown that Vitamin D deficiency has been shown to be able to down regulate Notch signalling (Domingues-Faria et al. 2014), providing robust confirmation of our finding in the current study. Further mechanistic study on how Vitamin D receptor signalling interacts with Notch signalling intracellularly will be important to further illustrate the cross talk of the two pathways. Fgf23, on the other hand, has not been reported to be linked with Notch pathway in any systems. Fgf23 signals through FGFR1 and Klotho (Razzaque 2009). the later has been proved to be a substrate of gamma-secretase (Bloch et al. 2009), which also cleaves Notch receptors, hence indicating a potential co-activation or suppression might persist in the Fgf23 and Notch pathways. It would be important to see in which level if and how they function simultaneously.

Previous work to advance knowledge about the crosstalk between different pathways has been on-going. Formerly we have found that in the dermal papilla cells, Wnt5a, a known canonical pathway member, exerts an important role in regulating Noggin, Fgf7 and 10 that ultimately control hair follicle keratinocyte fate (Hu et al. 2010). It is known that Wnt5a works through Frizzled receptors such as Frizzled 2, 3, 4 and 6 to activate calcium signalling or the non-canonical Wnt pathway (Kikuchi et al. 2012). The pathway obviously can affect body calcium level and be linked with Rickets. However very little has been known how it is functioned. Wnt5a plays an important role in the complex signalling cascade that coordinates chondrocyte growth and differentiation with subsequent osteoblast replacement and differentiation (Yamaguchi et al. 1999; Yang et al. 2003). In this study, we have found that in chondrocytes and osteoblasts expression of Wnt5a is also under RBP-Jkappa control and functional rescue experiments indicate that even in these cells Wnt5a is a key mediator of RBP-Jkappa function. In fact, treatment with this factor restored differentiation marker expression as well as that of other factors like Noggin and Ihh, which have a similar expression pattern as Wnt5a (Minina et al. 2001; Vortkamp et al. 1996; Yang et al. 2003) and exert a similarly essential function in bone development.

Taken together, our findings provide a potential novel and mechanistic explanation of Rickets symptoms have uncovered an essential connection between Notch function and control of Wnt5a expression, with a key master regulatory function in skeletal development.

## Materials and methods

### Mice

RBP-Jkappa loxp/loxp mice (Han et al. 2002) were crossed with transgenic mice expressing the Cre recombinase under control of the promoter/enhancer unit of the α2 chain of the collagen type I gene (Florin et al. 2004) to generate (ColIa2 Cre x RBP- Jkappa loxP/loxP) mice (Hu et al. 2012; Hu et al. 2010).

### Chondrocyte and osteoblast isolation

For chondrocyte isolation, the proximal growth plates of 20 tibiae from P10 mice (ColI-Cre x RBP-Jkappa loxP/loxP and RBP- Jkappa loxP/loxP controls) were dissected under a stereomicroscope. The dissected samples were washed twice in HBSS (Gibco), minced into small fragments, and digested in 20 ml 3% collagenase type I (Sigma) in HBSS for 1 hour at 37 °C with gentle shaking every 5 min. After the addition of 20 ml DMEM with 10% FBS, and filtering through double layer gauze to remove debris, cells were collected by centrifugation at 1000 r/min at 4 °C for 10 min.

For osteoblast isolation, frontal bones from 2 weeks old mice (10 mice per group) were dissected, washed twice in HBSS, and digested in 10 ml 0.25% trypsin, 1% collagenase, and 0.2% EDTA solution for 1 hour at 37 °C with gentle shaking every 5 min. The digested mixture was then mixed with 10 ml DMEM+ 10%FBS and filtered through double layer gauze to remove debris. Cells were collected by centrifugation at 1000 r/min at 4 °C for 10 min.

For both chondrocyte and osteoblast cultures, cell suspensions were depleted of CD34 positive cells (endothelial cells) by MACS prior to plating. Cells were plated at a density of 5 × 10^4^ per 60 mm dishes, and cultured in DMEM/F12 (Gibco) with 10% FBS until confluent (6 days for chondrocytes and 2 weeks for osteoblasts), before further analysis.

### Flow cytometry analysis

Flow cytometry was performed with conditions as were as previously described (Walker et al. 2019). Antibodies: CD24a and CD200 were purchased from BD biosciences. Osterix was purchased form Abcam. And Osteoculcacin was purchased from RnD systems.

### Recombinant protein treatment and beads implantation

Recombinant Wnt5a proteins were purchased from a commercial source (R&D systems) and dissolved in 1% BSA-PBS to achieve 100 μg/ml stock solutions. The final medium concentration of each these proteins for treatment of cultured cells was final concentration of 200 ng/ml.

### Bone and cartilage staining

For whole mount bone staining, P6 mice were sacrificed by cervical dislocation. After removal of skin and viscera, carcasses were fixed in 95% ethanol for 48 hours at room temperature. The samples were cleared in 2% KOH for other 24 hours. The skeletal system was stained with Alizarin red S (Fluka, 0.2% in water with 1% KOH) for 12 hours at room temperature. For cartilage staining, carcasses were stained in 0.5% Alcian Blue 8GX (Sigma, pH 2.5 in 20% acetic acid+ 80% ethanol) for 12 hours. The samples were stored in 100% glycerol for further examination.

For cartilage staining, the tibiae of 4 months old mice were isolated under the stereomicroscope and fixed in 4% paraformaldehyde in PBS (pH 7.4) at 4 °C overnight. After two 5 min washes in PBS, the tibiae were decalcified in 30 ml Osteosoft (Merck) for 8 days (with changes of Osteosoft every 2 days). Samples were then processed for paraffin embedding. Deparaffinized sections were stained in Alcian Blue 8GX for 1 hour at room temperature. Nuclear fast red stain was used for counterstaining (for 1 min).

### Micro-CT

High resolution micro-computerized tomography (micro-CT) (Skyscan 1076; Skyscan, Belgium) scanning of sacrificed mice was performed at a voxel size resolution of 9 μm. The 3D reconstruction was performed with the 3D modelling software “Nrecon”.

### Bone organ culture

Tibiae from P10 mice were isolated under sterile conditions and placed in 6-well culture plates (Costar, one tibia per well). Of the two tibiae recovered from each mouse, one was incubated with 2 ml culture medium (DMEM-10%FBS) plus 500 ng recombinant Wnt5a protein in a BSA solution, and the other with 2 ml culture medium with BSA solution alone. Tibiae were cultured for 7 days, with one change of medium and supplement at 2 days. Samples were fixed in 4% paraformaldehyde in PBS (pH 7.4) at 4 °C overnight. After two 5 min washes in PBS, the tibiae were decalcified in Osteosoft (Merck) and processed for paraffin embedding and Alcian Blue 8GX staining as described above. For quantification of cartilage thickness (including both proliferative and hypertrophic zones), measurements were taken along the proximal-distal axis using a calibration slide. The statistical significance of the results was calculated by paired T-test (using Prism 5 software).

### Quantitative real time RT-PCR, immunodetection and chromatin IP

Conditions for real time RT-PCR, and immunofluorescence (Hu et al. 2012). The list of gene-specific primers is provided in Supplemental Table 1. We used the following antibodies: Collagen Type I (GeneTex, 1:1000), Wnt5a (R&D systems, 1:200), Runx2 (SantaCruz, 1:200). Conditions for chromatin immunoprecipitation (ChIP) were as previously described (Walker et al. 2019).

## Supplementary Information

**Additional file 1: Supplemental Table 1.** Primers used in this study.

## Data Availability

All the data and materials are available from the corresponding author upon reasonable request.
