# Supplementary material for: Deletion of RBP-Jkappa gene in mesenchymal cells causes rickets like symptoms in the mouse
Source: Curr Med (Cham). 2022 May 26;1(1):7. doi: 10.1007/s44194-022-00007-w (PMC9177048; doi:10.1007/s44194-022-00007-w)
Supplement: Supplementary file 1 — Additional file 1: Supplemental Table 1. Primers used in this study. [file 44194_2022_7_MOESM1_ESM.docx]

**Supplemental Table 1. Primers used in this study.**

| AKP2 |  |  |
| --- | --- | --- |
| m.Akp2-F | TTG GGC AGG CAA GAC ACA GA | |
| m.Akp2-R | TTG GCA ACC CTG GGT AGA CAG | |
|  |  |  |
| Collagen 1 |  |  |
| m.ColIIa1_S3479 | GCA AAC AAG GAG ACA GAG GA | |
| m.ColIIa1_AS3687 | ACC CTG ATC TCC AGA AGG AC | |
|  |  |  |
| Collagen 2 |  |  |
| m.ColIIa1_S2077 | AAA GCT GGT GAG AAG GGT CT | |
| m.ColIIa1_AS2302 | CTC CAG CTT CAC CAG GAA TA | |
|  |  |  |
| Collagen X |  |  |
| m.ColX-F | AGA ACG GCA CGC CTA CGA T | |
| m.ColX-R | CCA TGA TTG CAC TCC CTG AA | |
|  |  |  |
| Indian Hedgehog |  |  |
| m.Ihh_S1932 | ATT CTT CAC ACG CAT TCC AT | |
| m.Ihh_AS2144 | CTG GCT GTG GTC TTT CAG TT | |
|  |  |  |
| Noggin |  |  |
| m.Noggin-F | TGTGGTCACAGACCTTCTGC | |
| m.Noggin-R | GTGAGGTGCACAGACTTGGA | |
|  |  |  |
| Osteocalcin |  |  |
| m.OC-F | GCA ATA AGG TAG TGA ACA GAC TCC | |
| m.OC-R | AGC AGG GTT AAG CTC ACA CTG | |
|  |  |  |
| Wnt5a |  |  |
| m.Wnt5a_S734 | GCTCCTGTAGCCTCAAGACG | |
| m.Wnt5a_AS821 | GCCGCGCTATCATACTTCTC | |

RBP-Jkappa

m.RBP-Jkappa F ACATCCATTACGGGCAGACT

m.RBP-Jkappa R TTTACGTGCGAGCACTTCTG
